# Engineering and characterization of interphases for lithium metal anodes

**DOI:** 10.1039/d1sc06181j

**Published:** 2021-12-08

**Authors:** Zulipiya Shadike, Sha Tan, Ruoqian Lin, Xia Cao, Enyuan Hu, Xiao-Qing Yang

**Affiliations:** Chemistry Division, Brookhaven National Laboratory Upton NY USA zshadike@sjtu.edu.cn enhu@bnl.gov xyang@bnl.gov; Energy and Environment Directorate, Pacific Northwest National Laboratory Richland WA USA

## Abstract

Lithium metal is a very promising anode material for achieving high energy density for next generation battery systems due to its low redox potential and high theoretical specific capacity of 3860 mA h g^−1^. However, dendrite formation and low coulombic efficiency during cycling greatly hindered its practical applications. The formation of a stable solid electrolyte interphase (SEI) on the lithium metal anode (LMA) holds the key to resolving these problems. A lot of techniques such as electrolyte modification, electrolyte additive introduction, and artificial SEI layer coating have been developed to form a stable SEI with capability to facilitate fast Li^+^ transportation and to suppress Li dendrite formation and undesired side reactions. It is well accepted that the chemical and physical properties of the SEI on the LMA are closely related to the kinetics of Li^+^ transport across the electrolyte–electrode interface and Li deposition behavior, which in turn affect the overall performance of the cell. Unfortunately, the chemical and structural complexity of the SEI makes it the least understood component of the battery cell. Recently various advanced *in situ* and *ex situ* characterization techniques have been developed to study the SEI and the results are quite interesting. Therefore, an overview about these new findings and development of SEI engineering and characterization is quite valuable to the battery research community. In this perspective, different strategies of SEI engineering are summarized, including electrolyte modification, electrolyte additive application, and artificial SEI construction. In addition, various advanced characterization techniques for investigating the SEI formation mechanism are discussed, including *in situ* visualization of the lithium deposition behavior, the quantification of inactive lithium, and using X-rays, neutrons and electrons as probing beams for both imaging and spectroscopy techniques with typical examples.

## Abstract 2

Different strategies of SEI engineering such as modification, additive application, and artificial SEI for electrolyte are summarized. Characterization techniques for SEI studies using X-ray, neutron, and electron as probing beams are discussed.

## Graphical abstract

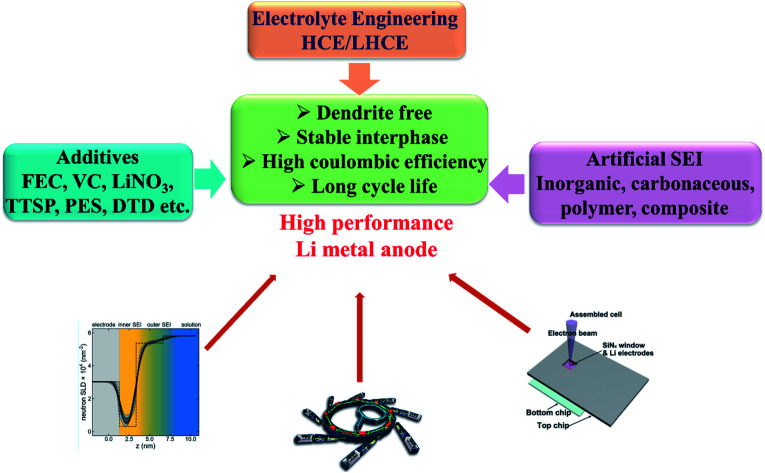

## 1. Introduction

The lithium metal anode (LMA) has been considered as the most promising anode for next generation devices owing to its high theoretical capacity (3860 mA h g^−1^), low density (0.534 g cm^−3^), and low electrochemical potential (−3.040 V *vs.* standard hydrogen electrode).^1^ However, the highly reactive lithium tends to react with organic electrolytes chemically and/or electrochemically. The insoluble electrolyte decomposition products are deposited on the Li anode, forming a solid electrolyte interphase (SEI). If the as-formed SEI is unstable during lithium plating/stripping, fresh Li will be exposed to the electrolyte and undesired side reactions will occur continuously, resulting in low coulombic efficiency (CE), short cycling life and lithium dendrite formation of lithium metal batteries (LMBs) and severely limited practical applications of LMBs. Therefore, designing a highly stable passivating SEI is critical in preventing the continuous side reactions between the LMA and electrolyte, regulating Li ion flux, and suppressing lithium dendrite formation.^2,3^ In LMBs, an ideal SEI should be electronically insulating and ionically conducting with good chemical/electrochemical stability to sustain long-term cyclability. In addition, mechanical properties, including the shear modulus and flexibility of the SEI, also play critical roles as well. To optimize the SEI properties, major efforts have been made through electrolyte engineering due to its low cost and high effectiveness. Electrolyte engineering not only involves the solvent, salt and additive selection, but also the solvation structure manipulation by controlling electrolyte concentration.^4,5^ Another approach is by coating artificial SEIs to obtain a controllable composition and structure.^6^

In recent decades, numerous achievements have been made regarding SEI engineering and the LMB electrochemical performance.^7^ At same time, continued efforts have been made for the fundamental understanding of this significant component, SEI. Since the first SEI model proposed by Peled^8^ in 1979, more models including the multilayer model, mosaic model and plum pudding model have been proposed and extensively investigated.^3^ However, many experimental observations could not be well explained by just a single model. Besides, the correlation between SEI composition, the structure and its passivation properties could not be fully understood yet. Therefore, research groups have been developing new advanced characterization techniques to study the composition and structure of the SEI.

In this perspective, SEI improvement methods including electrolyte engineering and artificial SEI construction are discussed first. The progress of advanced characterization techniques and their applications in understanding the formation and function mechanism of the SEI are discussed in the following section. Recent progress in employing synchrotron X-ray, neutron, cryo-electron microscopy (EM), time-of-flight secondary ion mass spectrometry (TOF-SIMS) and tomography techniques to study the SEI components and 3D morphology of lithium deposition are highlighted. In addition, new techniques for the quantification of inactive “dead” lithium and its contribution to the coulombic efficiency loss using titration gas analysis (TGA) and *in situ* X-ray diffraction techniques are discussed. At the end, prospects regarding future research on interphase engineering methods and advanced non-invasive characterization techniques are presented. We hope that this perspective could provide in-depth insights to the battery community and inspirations for future LMB development.

## 2. SEI engineering

### 2.1 Electrolyte engineering

To enable the stable cycling of the highly active LMA, research groups focus on SEI engineering by changing electrolyte formulations. Typical carbonate solvents used for lithium ion batteries (LIBs) (such as ethylene carbonate (EC) and ethyl methyl carbonate (EMC)) are highly reactive with Li metal, resulting in low coulombic efficiency (CE), Li dendrite formation and battery failure. Therefore, most of the reported electrolytes for the LMA today use ether as the solvent (1,2-dimethoxyethane (DME) and 1,3-dioxolane (DOL)), which is more compatible with Li metal. The widely used solvent DOL was reported to be able to form a stable polymerized surface inhibiting lithium dendrite growth.^9^ However, the poor oxidation stability of ether solvents limits their applications when coupled with high voltage cathodes (transition metal oxides). Therefore, different electrolyte modification strategies have been developed to improve the LMB performance.

#### 2.1.1 High concentration electrolytes (HCEs)

**Fig. 1:**
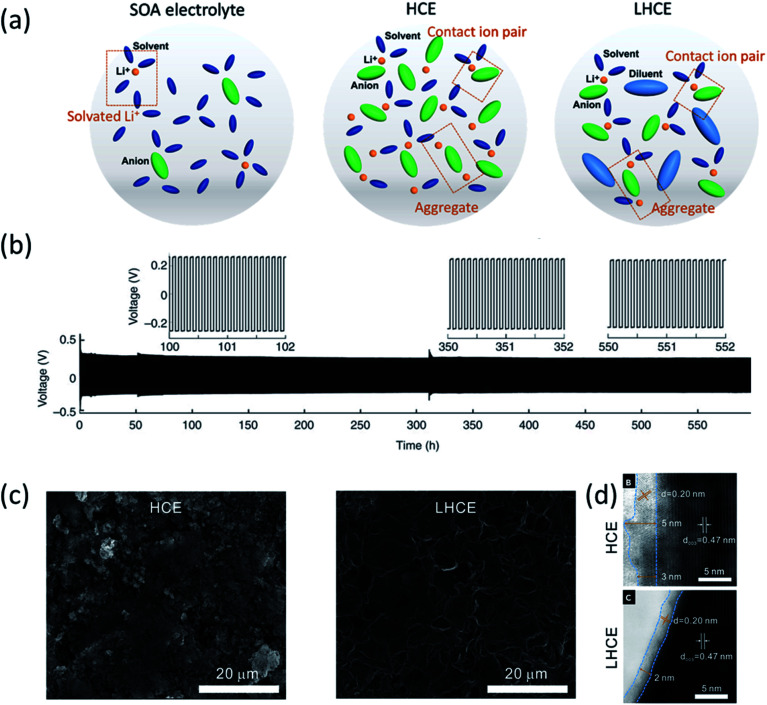
(a) Electrolyte solvation structures of state-of-the-art (SOA) electrolytes, high concentration electrolytes (HCEs) and localized high concentration electrolytes (LHCEs). Reproduced with permission.^5^ Copyright 2021 IOP Publishing Limited. (b) Li metal plating/stripping from a Li‖Li cell cycled at 10.0 mA cm^−2^ with a 4 M LiFSI-DME electrolyte. Reproduced with permission.^1^ Copyright 2015, Nature Publishing Group. (c) SEM images of the Li anode after cycling in Li‖NMC811 cells: 250 cycles in the HCE (LiFSI-1.2DME) and 300 cycles in the LHCE (LiFSI-1.2DME-3TTE). (d) ABF-STEM images of cycled NMC811 electrodes after 50 cycles at 4.4 V in the HCE and LHCE. Reproduced with permission.^17^ Copyright 2019, Elsevier Inc.

To maximize the ionic conductivity of LIB electrolytes, most of the commercial electrolytes have been using salt concentration around 1 M for decades. Ogumi *et al.*^10^ presented improved LMA performance in a highly concentrated LiTFSI/PC-based electrolyte and suggested that the deposition/dissolution behavior of lithium depends on the electrolyte concentration. Dendritic lithium growth was considerably suppressed and a thinner SEI was formed when the concentration of LiTFSI increased to 3.27 M. Yamada's group also made a lot of contribution to the development of HCEs for LIBs.^11,12^ They demonstrated ultrafast-charging capability of LIBs in 4.5 M LiFSA/AN and confirmed that such an improved performance should be attributed to the unique solvation structure of the super-concentrated electrolyte. The localized LUMOs at the TFSA^−^ anions suggest that it is mainly through the reduction of TFSA^−^ anions, rather than AN solvents, a stable SEI film was formed on a graphite anode. Recently, the application of HCEs has been reported as a very effective approach for the performance improvement of LMBs. When the salt concentration is increased, the content of free solvent molecules decreases, and the solvation structure changes, resulting in more contact ion pairs (CIPs) and aggregates (AGGs) as shown in Fig. 1a.^5^ The change of the solvation structure affects electrolyte redox behaviors. The CIP and AGG structures shifted the lowest unoccupied molecular orbital (LUMO) from solvent towards salt, resulting in a stable inorganic species (LiF, Li_2_O_*x*_)-rich SEI derived from the decomposition of salt anions. LiF is believed to be a critical SEI component for passivating Li metal because of its high bulk modulus (70 GPa), wide bandgap and high interfacial energy to lithium metal.^13^ Therefore, the LiF-rich SEI formed in HCEs enables stable cycling of LMBs even when carbonate and sulfonate based electrolytes were used, which are considered highly unstable with LMAs.^14,15^ HCEs could also be used to further improve the lithium metal passivation of ether electrolytes. For example, Zhang's group^1^ reported that when an electrolyte of 4 M LiFSI in DME was used, a high CE of up to 99.1% without dendrite growth in a Li‖Cu cell was achieved, and a long cycling life (>6000 cycles at 10 mA cm^−2^) was obtained using the Li‖Li symmetric cell as shown in Fig. 1b, demonstrating excellent lithium metal protection by the FSI^−^ anion derived SEI. Moreover, the highest occupied molecular orbital (HOMO) of HCEs is also shifted to a lower value, making the electrolyte more oxidative resistant. Xu and co-workers^16^ tested high voltage (>4.3 V) LMBs (Li‖NMC) using a HCE (4 M LiFSI in DME) electrolyte. A unique LiF-rich cathode electrolyte interphase (CEI) was formed to enable stable cycling of Li‖NMC batteries up to 4.5 V with high CE demonstrating the effect of the HCE on stabilizing the highly reactive NMC811 cathode surface at high voltages.

#### 2.1.2 Localized high concentration electrolytes (LHCEs)

Although HCEs significantly improved the oxidation stability and lithium metal passivation, the high cost and high viscosity of HCEs limit their practical applications. Therefore, low viscosity and low dielectric constant co-solvent, co-called “diluent”, is added into HCEs to form LHCEs. Generally, hydrofluoroethers (HFEs), such as 1,1,2,2-tetrafluoroethyl-2,2,3,3-tetrafluoropropyl ether (TTE), bis(2,2,2-trifluoroethyl) ether (BTFE) and tris(2,2,2-trifluoroethyl)orthoformate (TFEO), are used as diluents for LHCEs. The LHCE preserves the HCE solvation structure (Fig. 1a) and inherits the advantages of the HCE, while brings about better wettability and ionic conductivity at the same time. Xu *et al.*^17^ compared the electrochemical performances of Li‖NMC811 cells using a HCE (LiFSI–1.2DME (molar ratio)) and LHCE (LiFSI–1.2DME–3TTE (molar ratio)). In a cell of Li‖NMC811 using LiFSI–1.2DME–3TTE (LHCE), over 87% of its initial capacity after 250 cycles at a high cutoff voltage of 4.5 V was achieved, in comparison to the 76% retention using LiFSI–1.2DME (HCE). This significantly improved cycling performance was ascribed to the excellent Li metal protection so that a more uniform and integrated surface (Fig. 1c) was observed on the Li anode after cycling in Li‖NMC811 for 300 cycles. Furthermore, better cathode protection was demonstrated in the LHCE as presented in Fig. 1d, and a higher crystallinity and thinner CEI on NMC811 particles was generated when using the LHCE. In addition, the utilization of the LHCE can also extend LMB operating temperature to a wider range. Wang's group used a non-polar HFE as a diluent in a fully fluorinated electrolyte, and the as-formed LHCE enabled the stable operation of a Li‖LiNi_0.8_Co_0.15_Al_0.05_O_2_ (NCA) cell even at −85 °C and delivered ∼50% of its room temperature capacity.^18^ Although an anion derived interphase is the key to LHCEs, using different solvents, diluents and solvent/diluent ratios greatly affects the SEI/CEI formed and overall electrochemical performance. Zhang's group systematically studied the solvation structures and electronic structures using theoretical methods as well as experimental interphase characterization techniques, demonstrating the importance of solvent and diluents and their synergetic effects with the salt and the solvating solvent in designing LHCEs.^19–21^

#### 2.1.3 Fluorinated solvents and electrolytes

**Fig. 2:**
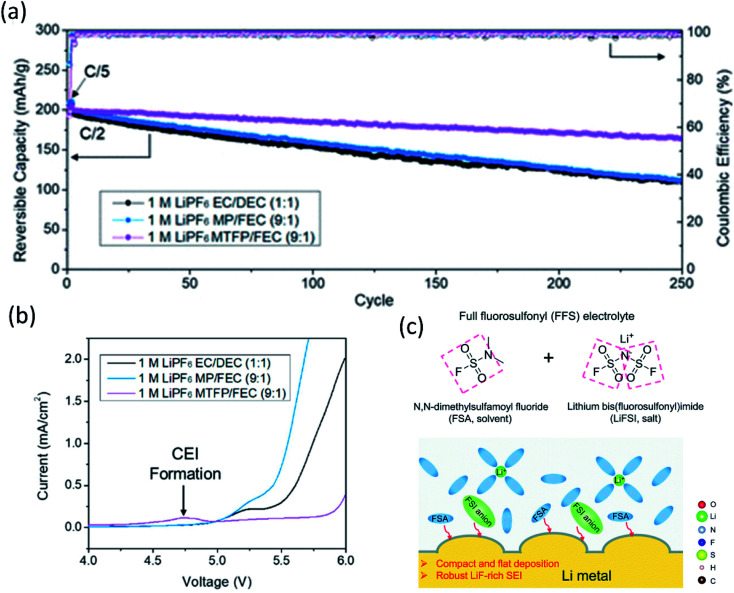
(a) Cycling performance of NMC 811‖Li half-cells at 0.5C. Reproduced with permission.^24^ Copyright 2020, American Chemical Society. (b) Linear Scan Voltammetry (LSV) profiles of conductive carbon electrodes at 1 mV s^−1^ in selected electrolytes: 1 M LiPF_6_ EC/DEC (1 : 1), 1 M LiPF_6_ MP/FEC (9 : 1), and 1 M LiPF_6_ MTFP/FEC (9 : 1) electrolytes. Reproduced with permission.^24^ Copyright 2020, American Chemical Society. (c) The FFS electrolyte is composed of FSA (solvent) and LiFSI (salt) with fluorosulfonyl groups in both components, and the schematic diagram of the Li growth and SEI formation mechanism in the FFS electrolyte. Reproduced with permission.^25^ Copyright 2020, from the Royal Society of Chemistry.

Apart from using HCEs/LHCEs, it's identified that solvent fluorination is another effective approach to improve oxidation stability^22^ and form a LiF-rich SEI.^23^ Carbonates and esters have high oxidation stability than ethers but suffer from instability with Li metal. So fluorinated carbonates, such as FEMC and FEC, were widely used as co-solvents for high voltage LMBs. Similarly, fluorinated esters were investigated as well. Chen *et al.*^24^ studied the electrochemical performance using 1 M LiPF_6_ in methyl propionate (MP), and in its fluorinated counterpart, methyl 3,3,3-trifluoropionate (MTFP). To investigate the Li metal protection, Li‖Cu cells were tested, and the CE of the MTFP electrolyte was surprisingly high, 97.6%, close to that of DOL/DME electrolytes, while the CE of the MP electrolyte was only 6.2%. This huge difference was ascribed to the great protection provided by the LiF-rich SEI formed in the MTFP electrolyte. The MTFP electrolyte also showed excellent electrochemical performance cycling in a Li‖NMC811 cell (2.8–4.5 V) as shown in Fig. 2a, which had a capacity retention of 80% after 250 cycles at C/2, revealing that fluorination not only enhanced the carbonate/ester combability with lithium metal but also improved the oxidation stability or the CEI properties. As shown in Fig. 2b, after fluorination, the oxidation window was extended to ∼5.7 V *vs.* Li/Li^+^, with a small peak at 4.75 V for CEI formation.

Inspired by the super stable FSI^−^ derived interphase formed in HCEs/LHCEs, solvent molecules bearing one or more fluorosulfonyl groups were expected to generate a LiF rich SEI. Therefore, dimethylsulfamoyl fluoride (Fig. 2c) was utilized as an electrolyte solvent by Li's group.^25^ Coupled with 2.5 M LiFSI and 0.2 M LiPF_6_, the “full fluorosulfonyl” electrolyte (FFS) provided surprisingly good protection of Li metal, yielded compact and flat Li deposition, and high stability on the cathode side, and thus the Li (60 μm thick)‖NMC622 cell retained 89% of its initial capacity after 200 cycles.

As mentioned before, ethers (*i.e.*, DME) are compatible with LMAs but have low oxidation stability. HFEs have extended the electrochemical stability window but suffer from low salt solubility. To combine the merits, Bao's group synthesized a new compound, fluorinated 1,4-dimethoxylbutane (FDMB), which combined the ether group and –CF_2_– segment to be able to solvate Li^+^ ions and form a stable SEI/CEI on LMA/cathodes (high voltage) at the same time.^26^ A unique Li–F interaction was observed in the FDMB electrolyte, which further leads to a FSI^−^ rich Li^+^ solvation sheath, thereby generating a LiF-rich interphase and inhibiting Al corrosion by FSI^−^. Therefore, Li‖Cu cells using 1 M LiFSI in the FDMB electrolyte reached a very high CE of 99.52%, and it can be stably cycled inside Li‖NMC batteries for 420 cycles with 90% capacity retention. This excellent electrochemical behavior indicated that the covalently bonding of ether and fluorinated segments can effectively combine the merits of both of them into one single molecule, providing a new way for solvent molecule design.

### 2.2 Additives

**Fig. 3:**
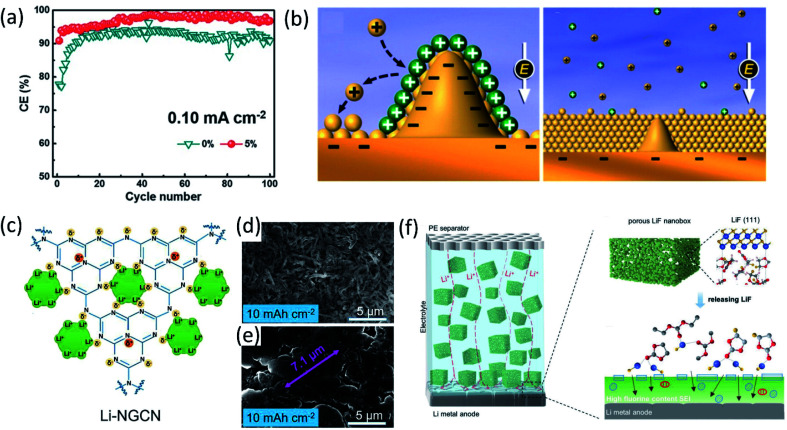
(a) CE at a current density of 0.10 mA cm^−2^ with a capacity of 0.5 mA h cm^−2^. Reproduced with permission.^28^ Copyright © 2017 WILEY-VCH. (b) Illustration of the Li deposition process based on the SHES mechanism. Reproduced with permission.^30^ Copyright © 2013, American Chemical Society. (c) Schematic diagram of Li-ion deposition on the NGCN layer. Top-view SEM of Li‖Li at an areal capacity of 10 mA h cm^−2^ using an electrolyte (d) without NGCN and (e) with NGCN. Reproduced (c–e) with permission.^36^ Copyright © 2021 Elsevier. (f) Schematic illustration of the merits of the electrolyte in porous LiF. Reproduced with permission.^37^ Copyright © 2021 Wiley-VCH.

To develop stable LMBs, another efficient approach is using additives to engineer electrolyte/electrode interphase formation for lithium dendrite suppression and CE improvement. Conventional electrolyte additives for LIBs such as vinylene carbonate (VC) can be used to protect LMAs as well. Yamaki's group demonstrated that the gel-like surface composed of polymeric VC reduction products can prevent the side reactions between lithium metal and electrolytes.^27^ Another extensively used additive is fluoroethylene carbonate (FEC). Zhang *et al.*^28^ added 5% FEC into a conventional carbonate electrolyte. Because of the lower LUMO energy of FEC, the resulting FEC-induced LiF-rich SEI prevented the reactions between carbonates and Li metal, forming uniform deposited lithium metal and improving coulombic efficiency to 98% in Li‖Cu cell as presented in Fig. 3a. Similarly, other organic additives used in LIBs such as tris(trimethylsilyl)phosphate (TTSP), prop-1-ene-1,3-sultone (PES), methylene methanedisulfonate (MMDS), and ethylene sulfate (DTD, also with the IUPAC name: 1,3,2-dioxathiolane 2,2-dioxide), were also investigated for Li metal protection.^29^ Inorganic species, for example, alkaline metal cations, were reported as additives for regulating Li metal deposition. In 2013, Xu, Zhang and co-workers proposed that alkaline ions (Cs^+^ or Rb^+^) can be used as additives for suppressing lithium dendrite growth through a self-healing electrostatic shield (SHES) mechanism.^30^ These cations have a lower reduction potential compared to Li^+^. During the lithium deposition process, the Cs^+^ accumulated around the deposited Li dendrites and formed a positively charged electrostatic shield around growing Li dendrites, repelling incoming Li^+^ and forcing deposited lithium to adjacent regions. Therefore, a self-smoothed film can be formed and the lithium dendrite formation can be suppressed (Fig. 3b).

Lithium nitrate (LiNO_3_) is widely used as an additive in ether electrolytes for Li–sulfur batteries. The inorganic species (such as Li_3_N and LiN_*x*_O_*y*_) generated by LiNO_3_ decomposition passivated the LMA and prevented its side reactions with electrolytes.^31^ Although LiNO_3_ has only limited solubility in carbonate solvents, the effects of LiNO_3_ on passivating lithium metal and regulating the deposited Li morphology in ether solvent can be extended to carbonate solvents as well. Cui and co-workers made a free-standing membrane composed of LiNO_3_ dissolved in a polymer matrix and attached on an anode, showing that the NO_3_^−^ in carbonate electrolytes regulated the Li nuclei morphology to spherical domains,^32^ resulting in a high average coulombic efficiency (∼98.1%) over 200 cycles. Using LiNO_3_ alone can sufficiently passivate lithium metal and improve the electrochemical performance, and the synergistic effects of using LiNO_3_ and other additives enhanced the lithium metal protection even further. Li *et al.*^33^ added both polysulfide (Li_2_S_8_) and LiNO_3_ additives into ether electrolytes (DOL-DME) for Li metal protection. LiNO_3_ reacted with lithium and passivated the LMA first and then the polysulfide reacted with lithium and formed a Li_2_S/Li_2_S_2_ containing SEI upper layer to minimize electrolyte decomposition. The synergistic effects of these two additives with lithium metal lead to the formation of a uniform SEI layer. Therefore, the Li‖Cu cell using an electrolyte containing both 0.18 M Li_2_S_8_ and 5 wt% LiNO_3_ maintains a high average coulombic efficiency (99.1%) from 100 to 400 cycles at a deposition current density of 1 mA cm^−2^. Likewise, Zhang's group proposed a new electrolyte containing both FEC and LiNO_3_, and the as-formed LiF and LiN_*x*_O_*y*_ containing SEI enabled a super high coulombic efficiency (99.6%) and ultralong cycle life (1000 cycles).^34^ However, it should be mentioned that, the protection provided by LiNO_3_ may not last long, because of the consumption of LiNO_3_ during repeated Li stripping/plating, limiting the long term cycling stability.^35^ Dispersing inorganic solids inside electrolytes as an additive has also been demonstrated as an effective approach. Bai *et al.*^36^ introduced nitrogen-defective graphite-like carbon nitride (NG-C_3_N_4_, or NGCN) into electrolytes by dispersing it into tetrahydrofuran (THF). During the initial electrochemical process, NGCN was deposited on the Li anode surface, forming an artificial SEI layer. As shown in Fig. 3c–e, the electronegative nature of pyridine and pyrrole nitrogen in NGCN caused edge adsorption of Li ions and regulated Li deposition. Recently, a porous LiF nanobox was added into an electrolyte for optimizing electrochemical performance.^37^ This highly porous LiF nanobox not only homogenized Li ion flux in the electrolyte, but also promoted fluorinated SEI formation by providing the fluorine source (Fig. 3f).

### 2.3 Artificial SEIs

In addition to the electrolyte formulation and additive application efforts to improve the as-formed SEI properties, the application of artificial SEIs is another effective approach. Depending on the composition, the artificial SEIs can be categorized into inorganic SEIs, carbonaceous SEIs, polymer SEIs and composite SEIs.

#### 2.3.1 Inorganic artificial SEIs

It was predicted that SEIs with a shear modulus higher than 6 GPa may sufficiently suppress lithium dendrite growth.^38^ Therefore, inorganic compounds with high mechanical strength (Al_2_O_3_, boron nitride, graphite nitride, diamonds, *etc.*) have been extensively investigated. Their chemical inertness and high shear modulus effectively suppressed dendrite formation. LiF, identified as the key component for LMA protection with a low in-plane diffusion barrier, has been widely used as an artificial SEI component. For example, Fan *et al.*^39^ deposited a LiF layer on Li or Cu foil using radio frequency magnetron sputtering.

**Fig. 4:**
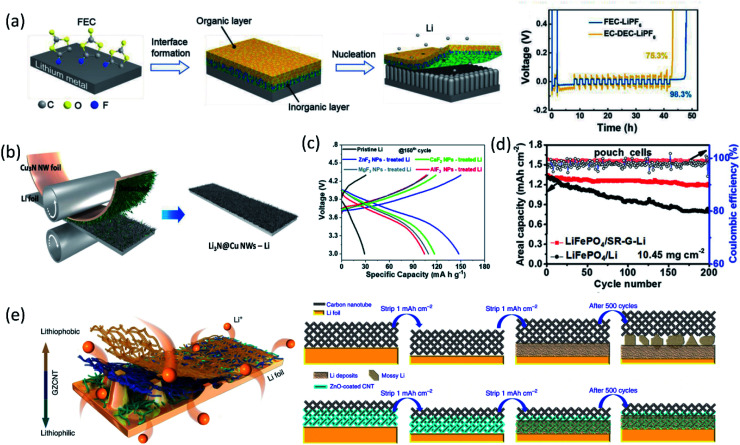
(a) Schematic diagram of the dual-layered film formation on a Li metal anode through FEC treatments; the voltage *versus* time plot in a CE evaluation test. Reproduced with permission.^40^ Copyright © 2018 WILEY-VCH. (b) Schematic illustration of Cu_3_N NWs printing onto bare Li foil by a facile roll-press method. Reproduced with permission.^45^ Copyright © 2020 WILEY-VCH. (c) Charge/discharge profiles of cells based on the pristine Li metal anode and MF_*x*_ NP-treated Li metal anodes in the 150th cycle. Reproduced with permission.^47^ Copyright © 2019 from the Royal Society of Chemistry. (d) The long-term galvanostatic cycling up to 200 cycles of the pouch cells of LiFePO_4_‖SR-G-Li and LiFePO_4_‖Li with a LiFePO_4_ loading of 10.45 mg cm^−2^ at 1C (1C = 170 mA h g^−1^). Reproduced with permission.^49^ Copyright © 2018 WILEY-VCH. (e) Schematic diagram for Li deposition of Li foil coated with a GZCNT interfacial layer; Li stripping/plating mechanism of Li foil coated with CNT and GZCNT interfacial layers. Reproduced with permission.^50^ Copyright © 2018, Nature Publishing Group.

With the passivation from the LiF layer, the coulombic efficiency was maintained at about 99% for more than 90 cycles using a PC electrolyte. Zhang and co-workers immersed Li metal foil directly into FEC solvent, formed a dual-layer structure with inorganic components (LiF and Li_2_CO_3_) at the bottom and organic species (ROCO_2_Li and ROLi) on the top *via* a spontaneous reaction between Li metal and FEC solvent.^40^ As shown in Fig. 4a, the inorganic rich bottom layer (∼50 nm) with a high mechanical modulus (7.0 GPa) can suppress Li dendrite growth, while the organic-rich top layer (∼25 nm) provided the flexibility and buffered the volume changes during the Li stripping/plating process, thereby regulating uniform Li deposition, preventing Li dendrite formation and significantly enhancing the CE of Li/Cu cells. Another study from Qiao's group created a chemically and mechanically stable artificial SEI composed of LiF, Sn and Sn–Li alloys by casting an electrolyte containing SnF_2_ on the lithium surface.^41^ The synergistic effect of these species inhibited the side reactions between Li metal and EC/DEC electrolytes and suppressed the Li dendrite growth, while facilitating ionic transfer and reversible Li–Sn alloying, enabling stable long-term Li‖Li symmetric cell cycling for over 2325 hours with a low overpotential.

Besides high mechanical strength, inorganics with high ionic conductivity were widely utilized as an artificial SEI as well. Guo and co-workers fabricated an artificial Li_3_PO_4_ SEI layer *via* an *in situ* reaction between polyphosphoric acid and Li metal.^42^ The as-formed Li_3_PO_4_ layer had a high Young's modulus (10–11 GPa), chemical stability and ionic conductivity, sufficiently suppressing the lithium dendrite growth and reducing the bulk Li corrosion after 200 cycles in the Li‖LiFePO_4_ cell. Through the reaction of Li metal with Li_2_S_6_ and P_2_S_5_, a single Li ion conductive layer, Li_3_PS_4_, was generated on the anode surface, which lowered the interfacial charge transfer resistance by 50-fold and maintained stable Li plating/stripping for 2500 hours in symmetric cells.^43^ Due to the high ionic conductivity (up to 10^−3^ S cm^−1^), an artificial SEI containing Li_3_N was also extensively studied. An artificial Li_3_N layer constructed by a reaction between Li metal and dry-N_2_ can provide a sufficient mechanical barrier to reduce lithium dendrite and regulated Li deposition.^44^ Recently, a Li_3_N@Cu nanowire (NW) layer was formed by simply printing Cu_3_N NWs onto bare Li foil *via* a facile roll-press method as shown in Fig. 4b, followed by a conversion reaction between Cu_3_N and Li.^45^ The synergetic effect of the chemical composition and structural uniqueness of the Li_3_N@Cu NWs-Li electrode greatly improved the Li plating behavior. The high Li^+^ conducting and electrically insulating Li_3_N and the unique 3D open channel structure regulated Li^+^ flux, leading to uniform and dense Li deposition at the bottom of the Li_3_N@Cu NW layer without Li dendrite growth. Constructing Li-rich Li-metal alloy films also can effectively prevent dendrite formation. For example, the direction reduction of metal chlorides by Li metal formed a Li_*x*_M alloy (M: In, Zn, Bi, As) film on the anode surface, allowing fast lithium diffusion.^46^ In addition, the by-product, LiCl, is electronically insulating, inhibiting further electrolyte decomposition. Similarly, a LiF/Li–M (M = Zn, Ca, Mg, Al) alloy artificial SEI layer was fabricated using MF_*x*_ nanoparticles for stabilizing LMAs.^47^ The synergistic effect between the formed LiF and LiM alloys suppressed Li dendrite formation and expanded the lifespan of Li‖NCA cells (Fig. 4c).

#### 2.3.2 Carbonaceous artificial SEIs

To accommodate continuous volume change during Li stripping/plating and avoid SEI breakage, artificial SEIs with flexibility are highly desirable. Therefore, carbon materials, which has low cost, adjustable morphologies, flexibility, and strong mechanical strength, were utilized as artificial SEIs. Cui *et al.* coated Li foil with a monolayer of interconnected amorphous carbon nanospheres with a high Young's modulus (∼200 GPa), which is mechanically strong enough to suppress Li dendrite growth.^48^ Also, the weakly bounded monolayer was able to move up and down to adjust the empty space during Li plating/stripping. Two dimensional (2D) reduced graphene oxide (rGO) was coated onto Li metal (SR-G-Li) to functionalize as a dendrite suppression layer and SEI stabilizer layer to avoid cell impedance build up. The cell exhibited excellent cycle life of 1000 cycles at a current density of 5 mA cm^−2^.^49^ Furthermore, a simple scalable spray-painting technique was developed, enabling simple fabrication of rGO coated Li foil and large-scale production of SR-G-Li‖LiFePO_4_ pouch cells. According to electrochemical measurements, the rGO coated Li foil enabled higher capacity retention and much better rate capability when coupled with a high mass loading (10.45 mg cm^−2^) of the LiFePO_4_ cathode (Fig. 4d).

Coupling carbon materials with other component to build composite artificial SEIs is another widely used strategy. Recently, Zhang *et al.*^50^ fabricated a lithiophilic–lithiophobic gradient interfacial layer composed of carbon nanotubes and a ZnO layer (termed GZCNT) on the LMA. According to electrochemical measurements, the GZCNT-coated cell maintained very low overpotential compared to bare Li foil and only CNT-coated Li for 1000 h. Especially, the GZCNT-coated Li kept excellent cycling behavior even at a high current density (10 mA cm^−2^). This great electrochemical performance was attributed to the unique two-layer structure. The bottom layer of lithiophilic ZnO/CNT tightly anchored to Li metal, enabling the formation of a uniform SEI, regulating Li deposition. Meanwhile, the top lithiophobic CNT has a porous structure, facilitated Li diffusion and avoided Li dendrite penetration owing to its mechanical strength (Fig. 4e).

#### 2.3.3 Polymer artificial SEIs

**Fig. 5:**
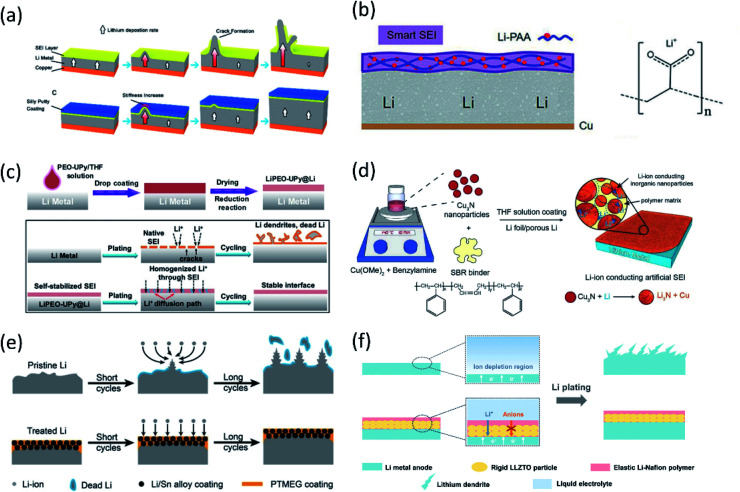
(a) Schematic diagrams showing the growth of Li dendrites for unprotected Li metal anodes. The covered dynamically crosslinked polymer (SP) can eliminate SEI cracking and potential catastrophic dendritic growth. Reproduced with permission.^52^ Copyright © 2017 American Chemical Society. (b) The design of the flexible SEI and the chemical structure of LiPAA. Reproduced with permission.^53^ Copyright © 2018 Wiley-VCH. (c) Schematic diagram of PEO–UPy coating on the Li metal surface. Li stripping/plating behavior for the bare Li and LiPEO–UPy@Li anodes. Reproduced with permission.^54^ Copyright © 2020 Wiley-VCH. (d) Schematic illustration of the fabrication of the Cu_3_N + SBR composite artificial SEI. Reproduced with permission.^55^ Copyright © 2016 WILEY-VCH. (e) Lithium deposition/stripping process using pristine Li and treated Li anodes. Reproduced with permission.^57^ Copyright © 2019 Wiley-VCH. (f) Schematic illustrations of different Li deposition patterns without and with a LLZTO-Nafion film. Reproduced with permission.^58^ Copyright © 2019 WILEY-VCH.

To further improve SEI flexibility and avoid SEI breakage due to volume changes, highly stretchable and flexible polymers are important artificial SEI candidates. *Via* spin-coating followed by HF etching, a poly(dimethylsiloxane) (PDMS) film with nanopores was coated on Li metal foil.^51^ Although a regular PDMS film is not a lithium conductor, the nanopores formed by acid treatment provided diffusion pathways for Li^+^ transportation. With this protective PDMS film, the lithium deposition morphology was regulated to suppress Li dendrite formation, thereby yielding a stable CE (∼95%) over 200 cycles in a conventional carbonate electrolyte. A boron cross-linked PDMS, Silly Putty (SP), was reported as a smart artificial SEI as well.^52^ Because of the “solid–liquid” properties owing to its dynamic covalent bonds, as shown in Fig. 5a, the SP layer can reversibly switch between its “solid” and “liquid” properties depending on the lithium growth rate during Li deposition/stripping to provide uniform surface coverage and suppress dendrite growth, in contrast to the as-formed Li filament upon deposition using an uncross-linked PDMS artificial SEI. Therefore, a high CE of 97% can be maintained over 120 cycles at 1 mA cm^−2^. Recently, Guo *et al.* fabricated a Li polyacrylic acid (LiPAA) SEI on Li foil *via* an *in situ* reaction between PAA and Li as shown in Fig. 5b.^53^ Normally, LiPAA polymer is used as an electrode binder due to its great binding ability and ionic conductivity, and these properties benefit the SEI as well. In addition, LiPAA is highly stretchable (582% strain to its initial length). With the LiPAA coating, the Li symmetric cell exhibited stable cycling for over 700 h at a current density of 0.5 mA cm^−2^. Co-polymer artificial SEIs were also studied because they can combine the merits of two different polymers. Xiong's group coated a self-healable supermolecular co-polymer composed of poly(ethylene oxide) (PEO) and ureido-pyrimidinone (UPy) segments on Li foil, forming LiPEO–UPy after a spontaneous reduction reaction with Li (Fig. 5c).^54^ The PEO segment is ionic conductive (2.37 × 10^−5^ S cm^−1^) and can homogenize the Li^+^ flux of Li plating/stripping due to the electrostatic interaction between the polar segments and Li^+^. In addition, the quadruple-hydrogen-bonding interactions of UPy dimers make it highly self-healable and stretchable. After coating PEO–UPy, long-term cycling life (∼1000 h) was achieved at a current density of 5 mA cm^−2^ and high capacity of 10 mA h cm^−2^. Besides the great protection over the Li anode, the polymer SEI also dramatically improved the electrochemical performance of the full cell, and the LiPEO–UPy@Li‖NMC622 cell had a higher capacity retention of 84.2% after 200 cycles at 1C in a voltage range of 2.8–4.3 V.

#### 2.3.4 Hybrid artificial SEIs

To combine the high mechanical strength of inorganic species and high flexibility of polymer, an inorganic/organic hybrid artificial SEI has been extensively investigated. Cui's groups designed an artificial SEI composed of Cu_3_N nanoparticles with styrene butadiene rubber (SBR) as shown in Fig. 5d.^55^ As mentioned before, a spontaneous reaction between Cu_3_N and Li generated highly ionic conductive Li_3_N, resulting in more uniform Li ion flux. The high mechanical strength, close packed inorganic nanoparticles suppressed Li dendrite growth. At the same time, the polymer, SBR, maintained the integrity of the SEI suppressing crack formation during Li deposition/stripping. The synergistic effect of these two components greatly increased CE (∼97.4% over 100 cycles) and regulated the uniform Li deposition. A similar strategy was reported by other research groups. A poly(vinylidenefluoride-*co*-hexafluoropropylene) (PVDF-HFP) and LiF hybrid SEI film with a thickness of 12 μm was deposited on Li metal.^56^ This protective film clearly improved the Li‖LiFePO_4_ cells with 2.5 times longer lifespan and high CE (>99.2%) in the cycling range. Coupling a lithiophilic alloy with polymer is another artificial SEI approach. Xie *et al.*^57^ constructed a poly(tetramethylene ether glycol) (PTMEG)–Li/Sn alloy hybrid layer (Fig. 5e). The symmetric cells using treated Li demonstrated a remarkably high cycling life (∼1000 h) with a relatively low overpotential. Plus, the Li–S cell and Li–LiFePO_4_ cell showed a highly improved capacity retention and lifespan. These enhanced electrochemical performances were attributed to the facile ionic transportation owing to the lithiophilic Li/Sn alloy with many Li vacancies and the PTMEG layer which has a strong affinity on Li. An ionic conductive, solid-state electrolyte also has been introduced into the artificial SEI. A garnet type solid state electrolyte component, Al doped Li_6.75_La_3_Zr_1.75_Ta_0.25_O_12_ (LLZTO), was combined with lithiated Nafion to functionalize as a dual-layer SEI (Fig. 5f).^58^ The mechanically strong and single-ion-conductive LLZTO bottom layer provided a physical barrier for dendrite suppression and rapid Li^+^ mobility. At the same time, the Li-Nafion top layer enables the interphase with enough deformability and robustness to accommodate electrode volume change. As a result, the deposited lithium underneath SEI layer in the symmetric cell still maintained a compact morphology and sustained a much longer lifespan compared with those for bare Li.

## 3. Advanced characterization techniques

To address the challenges of LMAs, various new characterization techniques have been developed to obtain in depth understanding of the interphasial chemistry as well as their effects on lithium deposition. In this section we will focus on several applications of advanced characterization techniques for interphase studies reported recently, rather than those conventional characterization techniques such as AFM, XPS, Raman, FTIR and EELS.

### 3.1 Electron microscopy (EM)

#### 3.1.1 *In situ* EM

**Fig. 6:**
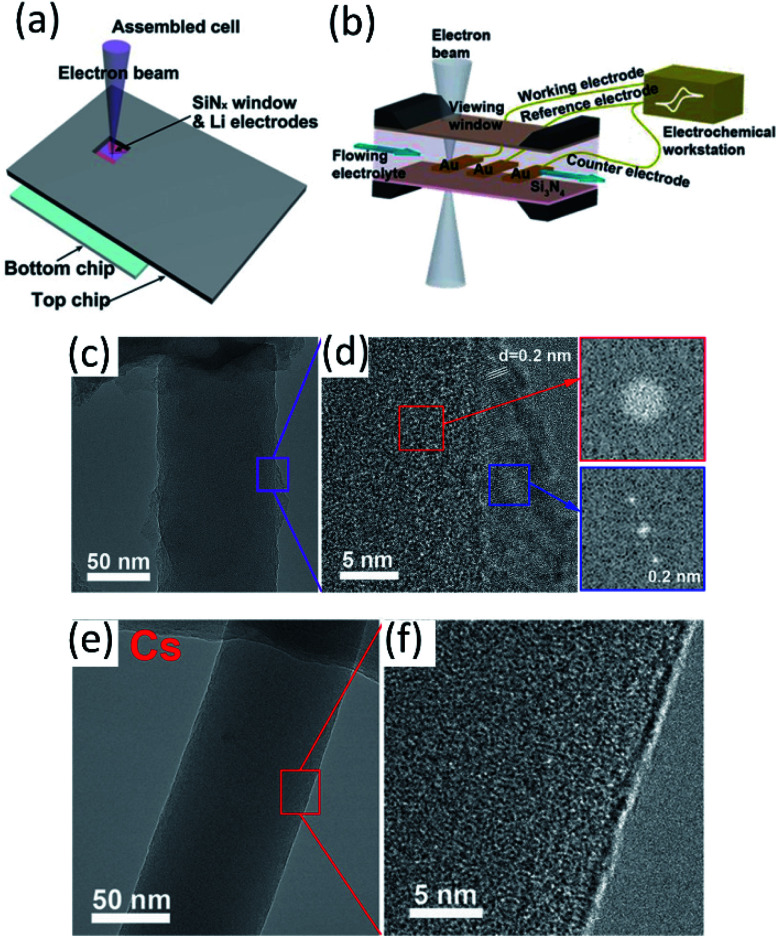
(a) Scheme of an *in situ* SEM-EC liquid cell (top view). Reproduced with permission.^59^ Copyright © 2017 WILEY-VCH. (b) The configuration of the lithium-ion microcell with three electrodes for electrochemical measurements. Reproduced with permission.^60^ Copyright © 2019 WILEY-VCH. Cryo-TEM image of EDLi using (c and d) a conventional carbonate electrolyte and (e and f) a Cs^+^ additive at 4 000 00× magnification. Reproduced (c–f) with permission.^63^ Copyright © 2017, American Chemical Society.

*Operando* visualization of electrode material evolution using EM during electrochemical cycling is a powerful technique to reveal structural, morphological and compositional changes. However, electron beam radiation damage to the electrolyte–electrode interphase and Li metal needs to be considered. *In situ* SEM was used to study the interphase in a Li‖Cu cell by Zhang's group.^59^ The lower electron beam dose of SEM ensured a more stable *operando* experiment compared to high dose *in situ* TEM, while providing higher resolution compared to optical microscopies. Using a specially designed *in situ* electrochemical SEM (EC-SEM) cell (Fig. 6a), Li could be clearly imaged through a SiN_*x*_ membrane view window, enabling the observation of Li dendrite growth, dissolution and “dead” Li formation during the plating/stripping process. The effects of LiNO_3_ and Li_2_S_8_ electrolyte additives on suppressing dendrite formation were also studied using this *operando* SEM technique. It is concluded that LiNO_3_ may facilitate the formation of a protective SEI and slow down the dendrite formation. However, the inhomogeneous dendrite growth indicates that such a SEI film is not uniform and LiNO_3_ alone is not effective enough to protect the lithium anode surface for long-term cycling. It was also reported that the Li dendrites could be chemically etched with a lithium polysulfide additive, leading to a smooth anode surface. *In situ* scanning TEM (STEM) was also used to study the formation, growth, and failure of a SEI at a high C rate (∼34C) using a lithium ion microcell as shown in Fig. 6b.^60^ It was observed that a typical bilayer structure (inner inorganic layer and outer organic layer) SEI was formed. During electrochemical charge, continuous SEI growth was detected, and the thickness of both layers followed a similar growth tendency, demonstrating radical assisted SEI growth after SEI thickness grown beyond the electron tunneling regime. During the discharge process, the rapid dissolution of an inorganic SEI layer after the SEI crack formation results in the delamination of the entire SEI film from the electrode. This work provides microscopic insights into the SEI structure and the dynamic evolution during the discharge/charge process to better understand SEI kinetics. *Operando* visualization of Li whisker growth using environmental TEM (ETEM) was reported by Wang and his co-workers.^61^ A specially designed setup using Li metal as the counter electrode coupled with a Ni coated AFM cantilever as the working electrode allows direct observation of Li nucleation when an electrical potential is applied. The Li nucleation and growth process was studied by ETEM inside different gas environments, which can control the formed SEI composition on the Li surface. In the CO_2_ gas atmosphere, a single crystal Li particle was initially formed. Afterwards, a Li whisker was sprout due to the lack of Li transport on the deposited Li surface. As a comparison, when Li nucleation took place in a N_2_ environment, the highly ionic conductive Li_3_N formed in the initial SEI promoted Li transportation, thereby yielding large Li particles without Li whisker formation.

#### 3.1.2 Cryo-TEM

To obtain high resolution imaging, high dose electron radiation is required, which would quickly deteriorate the radiation sensitive materials such as Li metal and the SEI. In addition, sample transfer could easily introduce contamination since both Li metal and the interphase are extremely chemically reactive. Therefore, cryo-TEM developed for structural biology was introduced for studying the Li metal deposition and interphase structures of LMBs with atomic level resolution. By coupling cryogenic sample transfer and measurement, the morphology and chemical information can be well preserved. Cui's group successfully observed single crystalline Li dendrites with different growing directions well-preserved under cryogenic conditions.^62^ Also, the SEI structure was studied. A mosaic layer formed in a conventional carbonate electrolyte was observed, while a more ordered multilayer SEI was obtained in the presence of a FEC additive. In the same year, Meng *et al.*^63^ reported their work on using cryo-EM to observe the nanostructure of electrochemically deposited Li (EDLi) and the interphase. They found that the initially deposited Li (5 min at 0.5 mA cm^−2^) was amorphous (Fig. 6c and d), which was partially covered with an uneven SEI distributed on the surface when using a conventional carbonate electrolyte (1 M LiPF_6_ in EC/EMC). After introducing some metal ions (Cs^+^) as additives into the electrolyte, a dense, uniform and ultrathin SEI (Fig. 6e and f) was observed, and fully covered EDLi, yielding higher coulombic efficiency. Besides the nanoscale morphology observation, composition characterization by energy dispersive X-ray spectroscopy (EDS) and electron energy loss spectroscopy (EELS) makes cryo-TEM even more powerful. By combining a cryo-focused ion beam (cryo-FIB) and cryo-scanning transmission electron microscopy (cryo-STEM), Kourkoutis *et al.*^64^ identified the existence of two different dendrites on the lithium anode with a distinct structure and composition. Particularly, the “type II” dendrite is mainly composed of LiH instead of Li metal. This brittle LiH was easily disconnected from the electrode and became “dead Li”, disproportionally contributing to capacity fading. In addition, they proposed that the fluorinated electrolyte system has excellent Li metal passivation because of not only LiF formation, but also LiH dendrite deficiency due to limited hydrogen in the electrolyte. Following the successful demonstration of cryo-TEM for Li metal and SEI characterization, it's widely used for studying the interphase formed under different conditions, including different current densities,^65^ high/low operation temperature,^66^ and with/without additives.^67^ Cryo-TEM has also been applied for investigating cathode electrolyte interphase (CEIs),^68^ solid-state electrolytes,^69^ and Na metal SEIs.^70^ Although the interphase is highly complicated and dynamic during electrochemical cycling, the morphology and composition information obtained from cryo-TEM can give researchers atomic scale understanding of the metal anode and its interaction with the electrolyte during cycling.

### 3.2 Nuclear magnetic resonance (NMR)

**Fig. 7:**
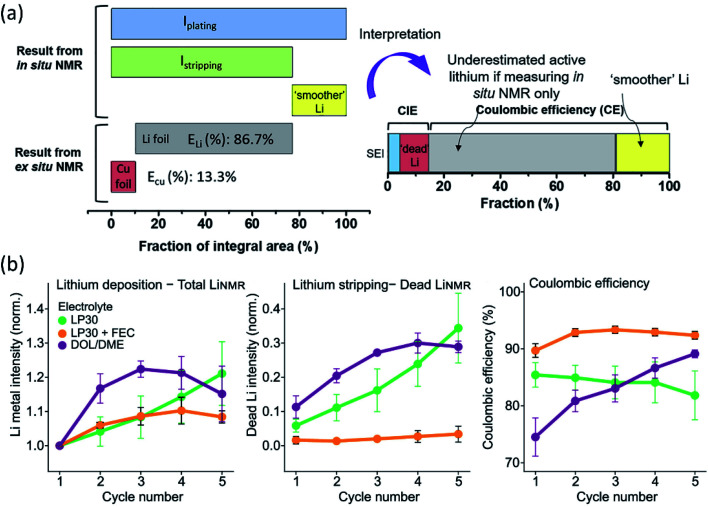
(a) Illustration of the integrated peak areas after one cycle of plating and stripping, as obtained from *in situ* and *ex situ*^7^Li NMR data. Reproduced with permission.^79^ Copyright 2020, Elsevier. (b) Average value of the normalized total LiNMR intensity at the end of plating, normalized dead LiNMR intensity at the end of stripping, and electrochemically obtained CE for the first five cycles in the three electrolytes, LP30 (green), LP30 + FEC (orange), and DOL/DME (purple). The error bars represent the standard deviation of the average values obtained in three different experiments. Reproduced with permission.^80^ Copyright © 2020, American Chemical Society.

As a non-destructive and quantitative method, NMR plays a critical role in LMB interphase characterization taking advantage of its solid/solution and *ex situ*/*in situ* capabilities. Multinuclear NMR methods provide valuable compositional information of the interphase. For example, by using 1D and 2D solution and solid-state NMR spectroscopy (^1^H, ^13^C, and ^7^Li), Wang *et al.*^71^ demonstrated that lithium ethylene mono-carbonate (LEMC) might be more likely the major SEI component for Li ion batteries using EC/DMC electrolytes rather than the widely accepted lithium ethylene di-carbonate (LEDC) reported in the literature. This LEMC benefits the Li^+^ transportation inside the SEI because of its high ionic conductivity (>1 × 10^−6^ S cm^−1^). Recently, another study from Yang's group identified the existence of NaH inside a SEI that was collected from Na‖Cu cells after 50 cycles by using *ex situ*^23^Na NMR.^72^ NaH and NaF passivated the Na metal and prevented further decomposition of the electrolyte due to their low electronic conductivity. Besides the insightful information of interphase composition provided by NMR, there are also reports using NMR to analyze the composition of electrolytes. In a specially designed *in situ* cell, electrolyte composition changes were monitored using NMR to study the electrolyte degradation during the electrochemical process.^73,74^ NMR was also utilized to analyze the collected electrolytes from cycled cells. Weber *et al.*^75^ demonstrated that the LiDFOB salt was consumed inside dual-salt electrolytes to form a SEI, stabilizing Li metal and achieving better electrochemical performance. Another significant aspect of NMR characterization is using solid state ^7^Li NMR to distinguish microstructured Li at the surface from the bulk. Because of the skin-depth effect, a radiofrequency field can only penetrate a limited depth (14.7 μm using a 4.7 T magnet) of Li, and microstructures on the surface can be detected. In general, bulk Li metal resonates at 240–250 ppm, while microstructure Li at the surface is at 260–270 ppm. Meanwhile, Li^+^ in the electrolyte and Li^+^ trapping inside the SEI is at ∼0 ppm. Therefore, Li in different environments can be easily detected by ^7^Li NMR. Using NMR to observe bulk Li and microstructure surface Li was first reported by Grey's group, and they used *in situ* solid state ^7^Li NMR to monitor the Li microstructure plating and stripping inside Li symmetric cells using different ionic liquid electrolytes.^71,76^ Following this, the impacts of the electrolyte additive, pressure, and separator in Li microstructure development were also studied by ^7^Li NMR.^77,78^ In addition, according to recent results, the microstructure morphology has an impact on NMR chemical shift as well. *In situ* and *ex situ*^7^Li NMR were utilized to perform quantitative Li loss analysis by Hsieh *et al.*^79^ In ^7^Li-NMR, signals for lithium ions within both the liquid electrolyte and the SEI appear at chemical shifts around 0 ppm, while metallic lithium due to Knight shifts has a higher ^7^Li-NMR chemical shift at about 250 ppm. Lithium deposits with mossy or dendritic morphologies different from (‘smooth’) bulk lithium and varying orientations with respect to the external magnetic field due to bulk magnetic susceptibility have higher chemical shifts in the range of 260–300 ppm. However, *in situ*^7^Li NMR could not capture all individual lithium fractions, underestimating the actual amounts of active but also dead or SEI Li fractions. To fully elucidate all individual contributions to the overall lithium deposits and their resulting ^7^Li NMR chemical shifts, an *in situ* cell was disassembled after the full stripping step, separating both the lithium and copper electrodes in a glove box, which were subsequently placed into pouch-type bags for complementary *ex situ* NMR measurements to determine the actual amounts of active lithium. Based on the CE from the electrochemical measurements of the Li‖Cu cell, the fractions of SEI formation, “dead Li” and “smoother Li” of the first cycle were obtained (Fig. 7a). Most of the *in situ* NMR studies were focused on Li‖Cu cells, and quantitative analysis of an anode-free cell Cu‖LiFePO_4_ was also feasible by utilizing *in situ*^7^Li NMR.^80^ This method successfully deconvoluted multiple capacity losses inside anode-free cells. Compared to additive-free carbonate electrolytes and ether electrolytes, carbonate electrolytes with FEC additives showed little “dead” Li formation for the first several cycles and high CE (Fig. 7b). Based on the obtained results of the three electrolyte systems, SEI formation contributed more to capacity loss than “dead” Li formation. The Li corrosion (Li metal dissolution) during the OCV period was monitored as well, showing that the suppression of such corrosion could be achieved by using polymer coated Cu foil or SEI passivation using electrolyte additives.

### 3.3 X-ray absorption and diffraction

As a most-developed surface sensitive technique, lab-based X-ray photoelectron spectroscopy (XPS) is commonly used to analyze SEI components qualitatively/quantitatively. XPS can provide the information about both inorganic and organic composition of SEIs due to the high elemental sensitivity, especially for light elements such as Li, C, N, F *etc.* In addition, the XPS technique can reveal the SEI composition variation from the top to the bulk *via* Ar^+^ sputtering. Lab-XPS analysis is frequently used to identify the SEI composition and the related results can be found in the literature. Conventional characterization techniques including XPS analysis for SEI study were comprehensively reviewed by Shan *et al.*^81^ and will not be discussed in this section. Synchrotron X-ray based techniques are powerful tools to study the SEI formation mechanism on LMAs due to the high flux and brilliance with a wide spectrum of X-ray beams which enables a variety of characterization capabilities including structural analysis, identification of newly formed phases and functional groups as well as determining the valence states of related elements. In recent years, synchrotron X-ray absorption, diffraction, and imaging techniques show unique capability in revealing the fundamental mechanism of SEIs and the stabilization effect of novel electrolytes, additives, and artificial SEIs.

**Fig. 8:**
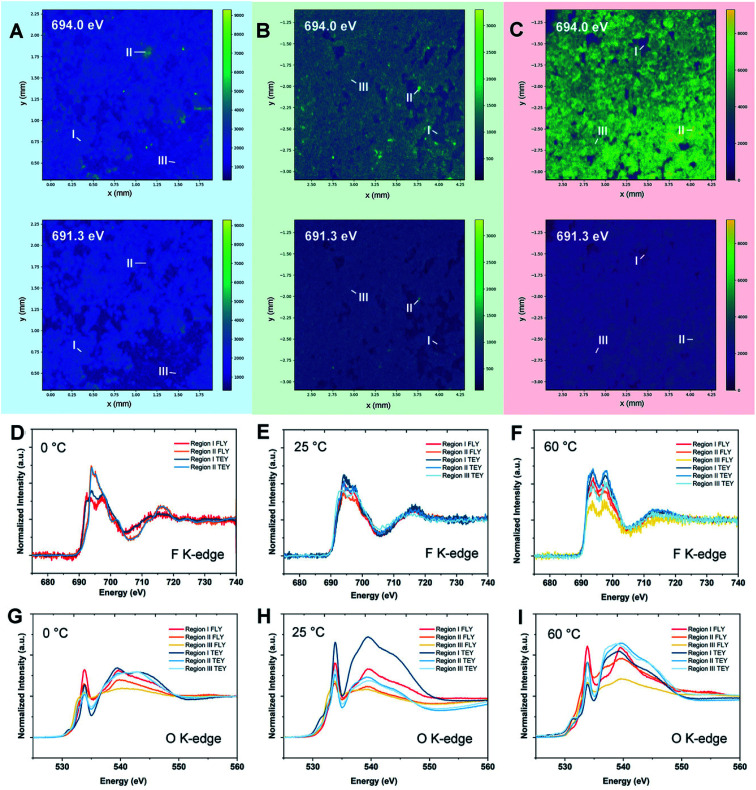
Synchrotron energy-dependent XRF mappings and micro-XANES measurements of the Li metal SEI at 0, 25, and 60 °C. XRF mappings of the Li metal surface after 10 cycles at (A) 0 °C, (B) 25 °C, and (C) 60 °C with incident energies of 691.3 and 694.0 eV. (D–F) F K-edge micro-XANES in regions I–III of Li metal cycled at 0, 25, and 60 °C, respectively. (G–I) O K-edge micro-XANES in regions I–III of Li metal cycled at 0, 25, and 60 °C, respectively. Reproduced with permission.^82^ Copyright © 2020 WILEY-VCH.

X-ray absorption spectroscopy (XAS) is a versatile technique that can provide information on the oxidation state and coordination environment of certain elements in crystalline and amorphous states. Taking advantage of the XAS technique which has high elemental selectivity, complicated SEIs can be studied by analyzing the chemical environment of different elements. Typically, SEIs contain inorganic components, such as Li_2_CO_3_, LiF, Li_2_O, Li_3_N, and LiOH and organic species. Therefore, soft XAS (SXAS) using incident photon energy less than 2 KeV is a good choice to study the composition of SEIs, which are mainly made of light elements. In addition, total electron yield (TEY) and fluorescence yield (FY) modes can provide surface and bulk SEI information, respectively. A successful example of SEI study using XAS was presented by Sun's group.^82^ Using energy dependent X-ray fluorescence (XRF) mapping, the inhomogeneous chemical composition of the SEI is visualized for cells cycled at different temperatures in carbonate electrolytes and is further coupled with micro-X-ray absorption near edge structure (micro-XANES) to provide information on SEI components present on the surface and at depth. XRF mapping is a chemically selective imaging technique for mapping the distribution of different chemical species. The incident energy used for collecting XRF images is tuned to the “fingerprint” spectral features in XANES. By controlling the energy of incident photons, the XRF mapping of different chemical species containing certain elements can be achieved. The normalized intensity of each spot in the XRF image then provides the relative concentration of that component at different parts of the electrode. XRF mappings at energies of 691.3 and 694.0 eV were presented in this work, which are associated with energies below and at the peak of the F 1s → unoccupied 2p state white line transition of LiF, respectively, with the results shown in Fig. 8. By analyzing F K-edge XAS and XRF mappings as well as O K-edge XAS, they directly visualized local variation in SEI formation, which cannot be achieved by the XPS technique since XPS can only provide average chemical information over a large area. Their result showed that the LiF formation in the SEI is inhomogeneously dispersed throughout the surface on the LMA cycled at 0 °C, with isolated regions having different bulk SEI species. For the LMA cycled at 25 °C and 60 °C, the spatial distribution of F species is more uniform across the entire surface, but still has certain differences in the chemical composition of the near surface region and bulk. Moreover, O K-edge XAS revealed that Li_2_CO_3_ is a major inorganic component of the bulk SEI and the ratio of carbonate to other organic species differs depending on the operating temperature. XRF mapping using a tender energy X-ray source was also applied to the Li-sulfurized polyacrylonitrile (SPAN) system to study the crosstalk of polysulfides and the additive effect on the Li deposition morphology of the LMA.^83^ XRF mapping not only showed the distribution of S contained SEI species but also exhibited the morphology of lithium metal underneath it. XRF images showed uneven Li deposition in carbonate-based electrolytes, but spherical and relatively uniform Li deposition in ether-based electrolytes. S K-edge XAS of selected spots proved polysulfide shuttling and formation of Li_2_S on the LMA as part of SEI components in ether-based electrolytes. The polysulfide shuttling was suppressed by adding the LiNO_3_ additive.

**Fig. 9:**
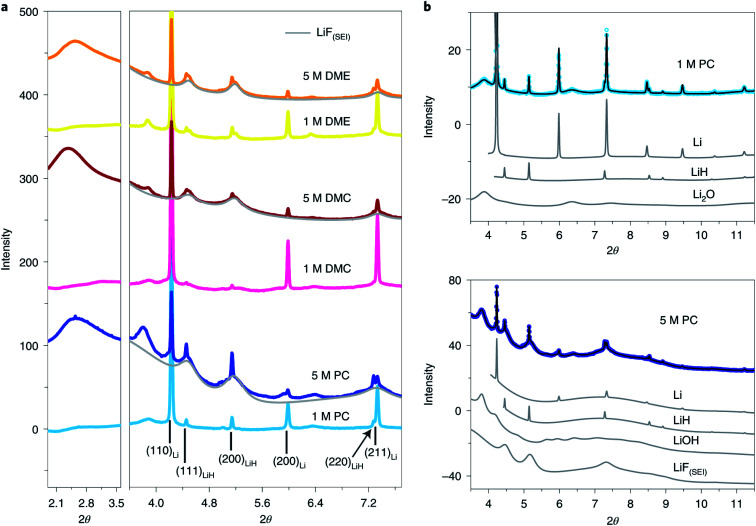
(a) SEI XRD of LCEs and HCEs using LiFSI as the salt and PC, DMC and DME as the solvents. The light grey pattern belongs to LiF(SEI). The wavelength used is 0.18323 Å. (b) Rietveld refinement of SEI XRD data of low- (upper) and high-concentration (lower) LiFSI in PC. Contributions of individual phases to the XRD pattern are also shown. Open circles, experimental data; black lines, calculated data. Reproduced with permission.^84^ Copyright © 2021, Springer Nature Limited.

In addition to the absorption technique, synchrotron XRD in one of the most attractive methods to achieve structural and chemical information of SEI components. However, the identification and quantification of the SEI using XRD face great challenges due to there being extremely small sample amounts and possible radiation damage as well as the complex nature of the SEI. Quantitatively studies of the composition of SEI on LMAs were reported by Shadike *et al.*^84^ SEI samples collected from LMAs cycled in different electrolytes were studied by synchrotron based XRD. In addition to the detection of several well-known components in the SEI, such as Li metal, Li_2_O and LiOH (Fig. 9), several important new findings were obtained by analyzing XRD patterns and Rietveld refinement results. Firstly, the Rietveld refinement result and *in situ* air exposure experiment of the SEI sample provided solid evidence for the existence of LiH as an important component in the SEI, which has been debated by research groups in recent years. LiH has the same crystal structure as LiF but slightly different lattice parameters making the differentiation of LiH from LiF quite difficult. The decomposition of LiH with moisture captured during *in situ* air exposure XRD clearly distinguished it from moisture stable LiF. Secondly, SEI-LiF was discovered to have nanocrystalline features with a grain size of around 3 nm, which is different from the regular bulk LiF with a much larger grain size. In addition, SEI-LiF has a larger lattice parameter than typical LiF, suggesting the possible formation of LiH_*x*_F_1−*x*_ solid solution. These results give a good answer to the question of why LiF can play a critical role in the formation of a good SEI while the regular bulk LiF is an ionic insulator: the nanocrystalline form and the larger lattice parameter both favor Li ion transport. Quantitative analysis revealed that SEI-LiF is in high abundance in SEI samples collected from high concentration electrolytes, in which fluorine-containing anions in the solvation sheath provide a fluorine source to form SEI-LiF and result in a high coulombic efficiency compared with low concentration electrolytes. These results demonstrated that the XRD technique is a powerful tool to identify new phases in the SEI and analyze its components quantitatively. However, XRD is suitable to study samples in crystalline phases but not those in amorphous phases, which are also the major components of the SEI. Pair distribution function (PDF) analysis is another advanced X-ray (or neutron) technique for SEI characterization. In PDF measurement, total scattering signals including both Bragg scattering and diffuse scattering are collected to provide atomic correlations regardless of the phase form (crystalline or amorphous).^85,86^ By utilizing this unique capability of the PDF technique, Shadike *et al.*^84^ further examined SEI components collected in different electrolytes with low and high concentrations. PDF results confirmed that Li_2_(FSI_(−F)_)_2_ (“−F” means one fluorine is removed), a decomposition product of LiTFSI salt, is the major amorphous phase in the SEI formed in high concentration electrolytes, while alkyl carbonates and Li_2_CO_3_ are the major amorphous components in the SEI from low concentration electrolytes.

### 3.4 X-ray imaging

To fully understand the interphases on LMAs, imaging the lithium deposition morphology including the features of lithium dendrites and SEIs is critically important. However, it is quite difficult to get the complex 3D morphology of the deposition layer using conventional imaging techniques. A range of imaging techniques have been developed previously to study the nature of lithium deposition in LMAs. Among them, optical microscopy is commonly used to show the 2D microstructure of the lithium deposition layer by an *ex situ* experimental setup, which requires cell dissembling after electrochemical cycling and removal of lithium microstructure samples from their as-grown environment. X-ray imaging is a nondestructive technique which provides 3D images *in situ* with high spatial and temporal resolutions, and therefore has been increasingly applied to study battery materials. The application of the transmission X-ray microscopy (TXM) technique for studying the Li deposition morphology has been reported in several publications. Eastwood *et al.*^87^ introduced *in situ* TXM for detecting lithium plating behavior. A specially designed *in situ* cell with a Kapton capillary as a body of the cell for X-ray penetration through the whole cell was used for *in situ* detecting Li deposition in a Li‖Li symmetric cell with a carbonate-based electrolyte. By applying the single-distance phase backpropagation algorithm to the projections to enhance contrast between the lithium (lower X-ray attenuation), electrolyte (intermediate attenuation) and lithium salt (higher attenuation) components, the generation of mossy lithium was successfully detected. More recently, Pan *et al.*^88^ employed micrometer-resolution X-ray computed tomography (XCT) with a low incident energy and a high-brightness liquid–metal–jet X-ray source to get good imaging contrast for Li plating. In their experimental set-up, the brightness of the X-ray source is approximately one order of magnitude higher than those of conventional microfocus Lab X-ray sources, enabling a tomography technique to achieve good data quality with a high signal-to-noise ratio and fine spatial resolution when used to study low-Z elements. Moreover, the feasibility of XCT for distinguishing the pore structure from deposited lithium was verified. To achieve phase contrast imaging, Sun's group applied an in-line coherent synchrotron X-ray source to investigate the influence of temperature on the macroscopic morphology of LMAs in 3D.^82^ Different phase contrasts of “cycled” and uncycled lithium layers were clearly shown in XCT imaging but the “inactive” lithium still can be distinguished from the deposited one because the surface is encapsulated by a SEI layer. Although, the X-ray imaging technique showed great potential in LMA study, it still needs more efforts to optimize the experimental set-up and data processing for studying SEI layers with good contrast for low-Z elements.

### 3.5 Neutron Reflectometry

Neutron Reflectometry (NR) is a versatile method which can probe the morphological and compositional changes in SEIs over a depth of up to ∼100 nm. Generally, a scattering length density (SLD) profile over the interface depth is revealed by analyzing and modeling specific modulations in the specular reflectivity of thermal neutrons as a function of momentum transfer upon scattering.^89,90^ Compared to X-ray reflectometry, NR in sensitive to the distribution of light elements such as H, Li, O and N since neutron scattering lengths vary in a nonmonotonic manner with the atomic number and with the isotope, which enables the determination of the SEI properties as a function of electrolyte composition. Owejan *et al.*^91^ pioneered the *in operando* NR studies of SEI layers as a function of potentials formed on copper electrodes with a titanium adhesion layer on silicon cycled in carbonate-based electrolytes (solvents were deuterated). The evolution of SEI thickness along with the cycle number can be clearly observed from NR results.

**Fig. 10:**
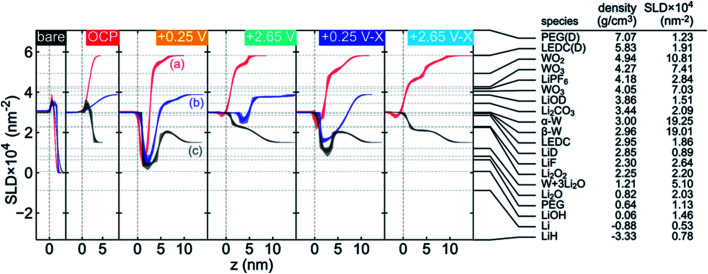
Comparison of the fitted SLD profile for each contrast at each applied potential (noted in the upper right of each plot, shown in the chronological order from left to right) and a list of SEI candidate species with the corresponding SLDs calculated from the listed literature densities. The SLD profiles have been offset along the *z* axis to facilitate comparison. The labels in the +0.25 V panel indicate (a) both solvents deuterated, (b) only DEC deuterated, and (c) both solvents at natural isotopic abundance. Two values are listed for the WO_3_ SLD to indicate upper and lower limits on reported densities (hexagonal WO_3_ excluded). LEDC is lithium ethylene dicarbonate and PEG is poly(ethylene glycol). Densities for the deuterated materials were calculated from densities reported for their natural abundance counterparts, assuming that the number density is unaffected by deuteration. Reproduced with permission.^92^ Copyright © 2019, American Chemical Society.

A series *in situ* isotopic labeling/contrast variation NR measurement was conducted on a tungsten film electrode to gain a deeper understanding of SEI formation.^92^ Modeling and fitting results confirmed the formation of a two-layer SEI at reduced potential, in which the thickness of the inner SEI layer was determined in the range of 2–3.5 nm, while for the outer SEI layer a thickness of 3–5 nm was estimated. For the first reduction to 0.25 V *vs.* Li/Li^+^, Li_2_O was identified as the major constituent in the inner SEI, while the outer SEI layer had a significant volume fraction of solution-filled porosity or a large fraction of solvent-derived species. Moreover, the structure of the SEI was potential-dependent as shown in Fig. 10. After charging at 2.65 V, the SLD of the inner layer increased due to the removal of Li, and the SLD of inner/outer layers showed lower contrast after cycling.

### 3.6 Secondary ion mass spectroscopy

**Fig. 11:**
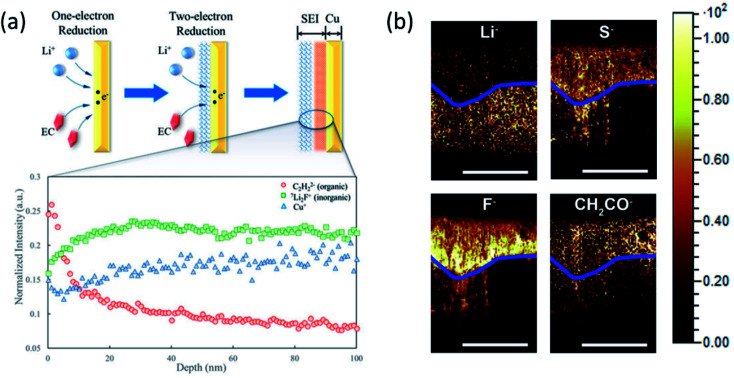
(a) ^6^Li:^7^Li ratio and Cu^+^, C_2_H_2_^−^ (organic), Li_2_F^−^ (inorganic), and Cu^+^. Intensities were normalized to total ion counts. Reproduced with permission.^93^ Copyright © 2018, American Chemical Society. (b) TOF-SIMS chemical mapping to indicate the Li^−^, S^−^, F^−^, and CH_2_CO^−^ species in the protective SEI layer of the Li anode. The blue line was artificially created to show the boundary of the reactive and residual Li. Scale bars represent 200 μm. Reproduced with permission.^96^ Copyright © 2017 Elsevier.

Secondary ion mass spectroscopy (SIMS) is an advanced surface characterization technique to provide elemental composition quantitatively on a surface or near surface. SIMS can accurately provide the SEI composition including all elements whether organic or inorganic and its sensitive is up to the ppm or even the ppb level. Therefore, SIMS has been widely used to study the molecular specific chemical properties of the species in electrode/electrolyte interphases formed on various anode and cathode materials and examine the effectiveness of additives and artificial SEI layers. A multilayer feature of SEIs with a compact inorganic inner layer and a porous organic outer layer is well accepted by battery community. Recently, to understand the SEI growth dynamics on a continuous time scale, Liu *et al.*^93^ developed an isotope-assisted time-of-flight SIMS (ToF-SIMS) approach as an *in situ* diagnostic method. In this study, a ^6^Li enriched electrolyte is used in the Li‖Cu cell and time-dependent SEI growth was recorded on the Cu electrode surface. During cycling, a ^6^Li containing electrolyte is continuously reduced to form a SEI and ^7^Li from the Li anode dissolves into the electrolyte to compensate for the Li^+^ ion consumption. With continuously formation of the SEI, the ^6^Li : ^7^Li ratio in the electrolyte would decrease over time. On the copper surface, the ^6^Li : ^7^Li ratio in the initially formed SEI would be higher than that of the subsequently formed SEI as shown in Fig. 11a. By analyzing ToF-SIMS results combined with XPS as shown in Fig. 11b, they proposed that the direct time sequence of SEI formation in different layers on the Cu surface follows a “bottom-up” mechanism: the organic components are formed first on the electrode; then, the inorganic components are formed underneath the organic layer and push the as-formed layer up as the SEI grows. This study lays a foundation of probing the real-time dynamics of the SEI layer on LMAs.

Also, the elemental distribution on the LMA surface was monitored and depth profiling was performed using ToF-SIMS to determine the concentration of the LiNO_3_ additive in the electrolyte by Wan's group.^94^ The results provided direct insights into the interfacial evolution at LMAs and the LiNO_3_-mediated mechanism. Besides, the composition of the artificial SEI layer and its uniform distribution on the LMA surface can also be explored by ToF-SIMS measurement combined with other imaging techniques.^95^ Another capability of ToF-SIMS demonstrated by Zhang's group is to probe element depth distribution in SEIs.^96^ ToF-SIMS mapping of major elements in SEIs such as Li^−^, S^−^, F^−^ and CH_2_CO^−^ organic groups in the cross section of LMAs clearly illustrated a dual structure including a reactive and residual lithium layer as shown in Fig. 11b. An important finding is that the signal of F^−^ species in reactive Li was much stronger than that in the residual Li, indicating the critical importance of LiF in the stable SEI. However, most of the measurements are conducted *ex situ* because the highly volatile liquid electrolyte in the *in situ* cell may evaporate under high vacuum in the chamber. Therefore, a specially designed *in situ* liquid cell is needed to capture intermediate phases and timely monitor SEI formation on LMAs in both static and dynamic modes.

### 3.7 Quantification of inactive lithium

It is well accepted that the low coulombic efficiency of LMAs is mainly caused by the repeated formation of the SEI layer and increased inactive lithium fraction. Inactive lithium is electrically and/or electrochemically isolated and composed of metallic lithium and Li^+^ which are no longer able to contribute to the energy storage process during charge–discharge cycling. However, these two different kinds of lithium cannot be easily distinguished since the metallic lithium might be surrounded by the SEI layer. Determining their roles in coulombic efficiency reduction of LMAs is critical to find an effective approach for improving the overall performance of LMAs. As mentioned in the previous section, the formation of the deposition layer can be visualized by the X-ray tomography technique, but it is not easy to quantify the inactive lithium. The titration gas chromatography (TGC) method was applied by Fang and Meng *et al.*^97^ to measure the amount of pristine and unreacted metallic lithium in different electrolytes and the calculation of the content of SEI-Li^+^. Combined with cryo-TEM analysis, they reported that the coulombic efficiency loss is governed by the formation of unreacted metallic lithium, significantly related to the electrolyte composition. They proposed effective approaches to improve coulombic efficiency including reactivation/minimization of the unreactive metallic lithium, optimization of the electrolyte, protection with an artificial SEI and application of a 3D host. More recently, Toney's^98^ group developed *in situ* synchrotron XRD with high flux to perform an *operando*, quantitative measurement of the amount of the Li metal on an electrode during the electrode plating and stripping process. Three different capacity loss mechanisms, such as electrolyte reduction, dead lithium formation, and corrosion, were successfully studies using this *operando* XRD.

## 4. Summary and prospects

(1) In this paper, three approaches for stable SEI engineering for LMAs are discussed: the new formulation of electrolytes, the application of additives, and the introduction of artificial SEIs. It is important to point out that the solvation structure of electrolytes profoundly determines the interphasial properties, and HCEs/LHCEs will continue to play major roles in the near future. As mentioned in the first section, HCEs enable the formation of anion derived SEIs (mainly inorganic/F-rich) due to the unique solvation structure. In addition to the stable SEI formation, low viscosity for facile Li^+^ diffusion is one of the great advantages of LHCEs. Although, LHCEs demonstrated various advantages in terms of improving the overall performance of LMAs, most of the fluorinated salts, solvents and diluents are more expensive than the conventional ones, due to the complex synthesis process. Therefore, the development of more cost effective chemicals and novel synthesis routes is needed. In addition to increasing salt concentration, altering solvent/salt combination can also change the solvation structure. Holoubek *et al.*^99^ prepared 1 M LiFSI in diethyl ether (DEE) as an electrolyte with a contact-ion pair (CIP) solvation structure leading to greatly improved LMA protection at room temperature and low temperatures compared with the 1 M LiFSI in DOL-DME electrolyte. A completely different approach than the high salt concentration in HCEs/LHCEs, results of low salt concentration at 0.4 M have been reported by Yu's group^100^ for good Li metal protection (CE of 97.6%) and stable Li‖LiFePO_4_ cycling (capacity retention of 95.4% after 300 cycles). These two examples provided alternative strategies to using HCEs/LHCEs for stable SEI electrolyte formulation, especially for low temperature application. Constructing artificial SEIs with designed structures and components is effective for LMA passivation. Taking multiple requirements for being a protective SEI layer into consideration, combining robust and ionic conductive inorganic species with a highly flexible organic layer is a more promising aspect. Apart from the protection nature of artificial SEIs, appropriate artificial SEI thickness without sacrificing energy density and simple scalable preparation methods should also be considered and optimized. In constructing artificial SEIs, the double layer structure of an inorganic inner layer and organic outer layer is an effective approach. The inorganic species have high ionic conductive and good mechanical strength while the organic layer is highly flexible against the volume change of LMAs. Anode engineering is one of the effective approaches for improving the electrochemical stability of LMAs. For example, the high specific area of 3D conductive hosts can homogenize the local current density and buffer Li^+^ flux, resulting in a dendrite free deposition of lithium. However, the loading amount of Li and the thickness of the 3D host for LMAs should be tailored carefully to achieve high energy density.

(2) For the characterization studies of SEIs, it is getting more and more important to use the combination of different advanced techniques for the same set of SEI samples to overcome the limitation of using a single technique alone (Table 1). For example, imaging techniques can provide the morphological features of lithium deposition layers but not the quantitative analysis of the composition of SEIs. When imaging techniques are combined with spectroscopic techniques, more completed information of SEIs is provided. A good example is the SEI study using the combination of cryo-TEM (imaging) and titration gas chromatography (spectroscopic) techniques.^97^ XRD is capable of providing the structural information of bulk SEIs but limited to the crystalline phase only. By combining XRD with PDF, which has the capability to study amorphous phases, both crystalline and amorphous phases in SEIs can be analyzed.^84^ We expect that the trend of using the combination of different characterization tools will continue in the future and multidimension and multimodal information will be reported. In addition, we expect that more efforts will be made towards the development of *in situ*/*operando* analytical techniques enabling the capability to study the evolution of SEIs continuously under realistic operating conditions.

**Table 1:** Capabilities and limitations of different characterization techniques

Characterization Techniques	Capabilities	Limitations
*In situ* EM	High spatial resolution for structure and morphology study	Special cell design is required for *in situ* experiments
	*In situ* observation of dynamic morphology changes	Possible radiation damage to the sample
Cryo-EM	High resolution down to the atomic level	Limited resource availability
	Able to obtain elemental and chemical information coupled with EELS and EDS. Preserve sensitive samples under cryogenic conditions (avoid radiation damage)	*In situ* experiments are quite difficult
NMR	Capable of detecting solid/liquid samples in *ex situ*/*in situ* modes	Spatial resolution is around the micro-level
	Capable of obtaining composition and microstructure information	
	Quantitative information of different Li morphologies	
SXAS	Capable of providing chemical and elemental information quantitatively	High vacuum or inert atmosphere is needed for measurement
	Chemical mapping of different SEI species coupled with XRF	
	Sensitive to light elements	
XRD	Provide the structure and chemical information of bulk SEIs quantitatively	Data analysis is difficult due to the complexity of SEIs
		Only provide the information of components of SEIs
PDF	Provide the structural information of both amorphous and crystalline phases of bulk SEIs quantitatively	Data analysis is difficult due to the complexity of SEIs
		Difficult to conduct *in situ* measurement
TXM	Capable of obtaining 2D/3D Li microstructure information	Complicated experimental setup and data process
	Capable of performing *in situ* measurement	
NR	Capable of obtaining morphological and compositional information at the top ∼100 nm	Limited resources
	Sensitive to light elements inside SEIs	Complicated data analysis process
SIMS	Surface sensitive	Need standard samples for quantification
	Provide compositional information at the ppm ppb^−1^ level	High energy beam would generate secondary products
	Capable of obtaining *in situ* visualization of SEI microstructures	

The composition and morphology of SEIs dynamically change during charge–discharge cycling and the ability to monitor such evolution will provide critical information about the stability of SEIs for long term cycling. Chemical and elemental sensitive soft XAS has shown great potential in interphase study and widely applied to identify the components in cathode/electrolyte interphases, but rarely used for SEI study on LMAs. Through the analysis of soft XAS at the K-edges of Li, O, C, N, and F, full patterns of both inorganic and organic species can be obtained. By analyzing data collected in total electron yield (TEY) and fluorescence yield (FY) modes, the components of surface and bulk SEIs can be determined, respectively. However, it is important to minimize the beam damage on SEI components by controlling the beam flux level to balance the high quality XAS spectra and tolerable flux for the reliability of XAS data.

Some new advanced techniques have been introduced for LMA studies recently. Synchrotron high energy XPS (HEXPS) is one of them with the capability to semi-quantitatively probe the depth-related evolution of SEI components through tuning the incident X-ray energy. Another example is the *in situ* liquid-SIMS technique with a special *in situ* cell which can be used in a high vacuum chamber and used for dynamic chemical mapping of the interfacial species. As reported by Zhu *et al.*^101^ a complete picture of the structure and composition of the electric double layer as well as its link to interphasial chemistry in LIBs was revealed by liquid-SIMS.

(3) We expect that the studies on LiH in SEIs will be getting more attention. The presence or absence of LiH has been debated for a long time.^64,97^ The possible formation of LiH in SEIs was first proposed in 1999,^102^ with the first experimental evidence reported in 2018^64^ using cryo-STEM mapping. However, the formation of LiH has remained controversial, as evidenced by multiple publications arguing no LiH observations in SEIs,^62,97^ including one that used a quantitative titration method.^97^ However, a new study^84^ using synchrotron-based XRD and PDF analysis provided new evidence about the presence of LiH in SEIs on LMAs. A separated study of Cui *et al.*^103^ reported the LiH and metallic Li contents of a cycled LMA determined by the combination of deuterium-oxide (D_2_O) titration experiments in an on-line gas analysis mass spectrometry (MS) system. They also claimed that the amount of LiH accumulation is negatively correlated with the cyclability of practical LMBs. However, the LiH content reported in this work is from the cycled cell not the SEI only. Therefore, it will be quite interesting to further study whether the effect of LiH content in the SEI is positive or negative on the cyclability, as well as the formation mechanism and the hydrogen source (solvent decomposition or H_2_O in the electrolyte) of LiH.

(4) Most of the mechanism studies on SEIs were conducted during the initial deposition process. However, for LMAs with unstable interphases, every cycle can be considered as the formation cycle due to the repeated formation of “fresh Li” and “new” SEIs. Achieving in-depth insight into the dynamic evolution of SEI formation is also an important direction for the rational design of stable SEIs. In addition to SEI engineering and advanced characterization techniques, theoretical study is essential for better understanding the relationship between the solvation structure of the electrolyte and SEI components. Calculation results could also provide guidance for selecting and screening electrolyte components and additives. In addition, electrolyte decomposition products and the formation mechanism of SEIs can be predicted by a theoretical study.

In summary, we believe that the studies of solid electrolyte interphases on the surface of Li-metal anodes will continuously play a critical role in enhancing our fundamental understanding of the interphase chemistry in batteries and providing valuable information for the development and improvement of LMBs.

## Author contributions

Z. Shadike, E. Hu, and X.-Q. Yang designed the scope of the paper. S. Shadike, S. Tan, R. Lin, X. Cao, E. Hu, and X.-Q. Yang wrote the paper.

## Conflicts of interest

There are no conflicts to declare.

## Supplementary Material
